# Investigation on Abnormal Iron Metabolism and Related Inflammation in Parkinson Disease Patients with Probable RBD

**DOI:** 10.1371/journal.pone.0138997

**Published:** 2015-10-02

**Authors:** Yang Hu, Shu-Yang Yu, Li-Jun Zuo, Ying-Shan Piao, Chen-Jie Cao, Fang Wang, Ze-Jie Chen, Yang Du, Teng-Hong Lian, Gai-Fen Liu, Ya-Jie Wang, Piu Chan, Sheng-Di Chen, Xiao-Min Wang, Wei Zhang

**Affiliations:** 1 Department of Geriatrics, Beijing Tiantan Hospital, Capital Medical University, Beijing, 100050, China; 2 Department of Neurology, Beijing Tiantan Hospital, Capital Medical University, Beijing, 100050, China; 3 China National Clinical Research Center for Neurological Diseases, Beijing, 100050, China; 4 Center of Parkinson's Disease, Beijing Institute for Brain Disorders, Beijing, 100053, China; 5 Beijing Key Laboratory on Parkinson Disease, Beijing, 100053, China; 6 Core Laboratory for Clinical Medical Research, Beijing Tiantan Hospital, Capital Medical University, Beijing, 100050, China; 7 Department of Neurology and Neurobiology, Beijing Institute of Geriatrics, Xuanwu Hospital of Capital Medical University, Beijing, 10053, China; 8 Department of Neurology, Ruijin Hospital Affiliated to Shanghai Jiaotong University School of Medicine, Shanghai, 200025, China; 9 Department of Physiology, Capital Medical University, Beijing, 100069, China; Oasi Institute for Research and Prevention of Mental Retardation, ITALY

## Abstract

**Objective:**

To investigate potential mechanisms involving abnormal iron metabolism and related inflammation in Parkinson disease (PD) patients with probable rapid eye movement sleep behavior disorder (PRBD).

**Methods:**

Total 210 PD patients and 31 controls were consecutively recruited. PD patients were evaluated by RBD Screening Questionnaire (RBDSQ) and classified into PRBD and probable no RBD (NPRBD) groups. Demographics information were recorded and clinical symptoms were evaluated by series of rating scales. Levels of iron and related proteins and inflammatory factors in cerebrospinal fluid (CSF) and serum were detected. Comparisons among control, NPRBD and PRBD groups and correlation analyses between RBDSQ score and levels of above factors were performed.

**Results:**

(1)The frequency of PRBD in PD patients is 31.90%. (2)PRBD group has longer disease duration, more advanced disease stage, severer motor symptoms and more non-motor symptoms than NPRBD group. (3)In CSF, levels of iron, transferrin, NO and IL–1β in PRBD group are prominently increased. RBDSQ score is positively correlated with the levels of iron, transferrin, NO and IL–1β in PD group. Iron level is positively correlated with the levels of NO and IL–1β in PD group. (4)In serum, transferrin level is prominently decreased in PRBD group. PGE_2_ level in PRBD group is drastically enhanced. RBDSQ score exhibits a positive correlation with PGE_2_ level in PD group.

**Conclusions:**

PRBD is common in PD patients. PRBD group has severer motor symptoms and more non-motor symptoms. Excessive iron in brain resulted from abnormal iron metabolism in central and peripheral systems is correlated with PRBD through neuroinflammation.

## Introduction

Parkinson disease (PD) is one of the most widespread neurodegenerative disorders with typical motor symptoms and a variety of non-motor symptoms. Rapid eye movement (REM) sleep behavior disorder (RBD) is a common non-motor symptom of PD, which prevalence is from 25% to 60% in PD population [1. Clinically, RBD is characterized by violent dreams and the action out of dream content during REM sleep [2], which may cause serious injury to both PD patients and their sleep partners. PD patients with RBD is associated with non-tremor-predominant subtype [3], severer autonomic dysfunctions [4], depression [3] and hallucinations [4], implying that PD patients with RBD may have more widespread and different underlying pattern of neurodegeneration. However, few investigations focus on the potential etiology and mechanism of RBD in PD patients.

Increasing studies show excessive iron depositions in substantia nigra (SN) and other nucleus in PD patients [5,6]. Iron-related neurodegeneration can be attributed for the defects in its metabolism and/or homeostasis and subsequent accumulation in the specific brain regions. For example, the level of transferrin, an iron metabolism-related protein, in brains of PD subjects is remarkably increased comparing with normal control subjects [7], indicating that abnormal iron metabolism in brain participate in the pathogenesis of PD. However, the levels of iron and related proteins, such as ferritin, in cerebrospinal fluid (CSF) or serum were not different comparing with healthy control subjects reported by previous investigations [8,9]. Moreover, no study detects the levels of iron and related proteins in CSF and serum in PD patients with RBD, and no investigation focuses on the correlation between RBD and iron metabolism in CSF and serum in PD patients.

Inflammation is a prominent feature of neurodegenerative disorders, which pathophysiologic role in the development and deterioration of PD has recently become a focus of investigation [10]. Inflammation in SN is characterized by over-activation of microglia and plenty of production of inflammatory factors, which causes dopaminergic neuronal death [11,12]. The dead neurons release cellular content, such as iron, into the extracellular spaces and aggravate inflammation by activating surrounding microglia, then propagate the degeneration of dopaminergic neurons and subsequent deterioration of motor symptoms of PD [13]. Recent studies have demonstrated that inflammation extends beyond SN and is related to non-motor symptoms of PD [14]. Additionally, it has been observed that peripheral inflammation may be relevant to neuroinflammation in brain [15] and play a potential role in the pathogenesis of non-motor symptoms of PD [16]. However, the role of inflammation in brain and peripheral system on RBD in PD patients remains unclear. Moreover, the relationship between iron and related proteins and inflammation in PD patients with RBD is unknown.

In this study, in PD patients, we assessed probable RBD (PRBD) by RBD Screening Questionnaire (RBDSQ), detected the levels of iron and related proteins, including transferrin, ferritin and lactoferrin, and inflammatory factors, including nitric oxide (NO), hydrogen peroxide (H_2_O_2_), interleukin (IL)-1β, prostaglandin (PG) E_2_ and tumor necrosis factor (TNF) -α in CSF and serum, and analyzed the correlations among RBDSQ score and the levels of above factors, so as to figure out the potential underlying mechanisms of PD with RBD relating iron metabolism and inflammation.

## Methods

This study was approved by Beijing Tiantan Hospital review board. Written informed consents were obtained from all participants in this study.

### 1. Subjects

#### 1.1 PD patients

Patients were diagnosed with PD according to United Kingdom Parkinson’s Disease Society Brain Bank criteria [17]. Total 210 PD patients were consecutively recruited from the Department of Geriatrics and the Department of Neurology, Beijing Tiantan Hospital, Capital Medical University from May 2010 to December 2013. Patients’ demographics information, including gender, age, age of onset, disease duration, disease severity, clinical phenotype, numbers of non-motor symptoms and levodopa equivalent does (LED) were recorded.

PD patients with blood donation histories, systemic diseases, including anemia, hepatosis, heart failure, pulmonary disorders and chronic renal failure were excluded. Female patients who had not been through menopause were not included in this study. PD patients with history of restless leg syndrome (RLS), periodic limb movement disorder (PLMD) and excessive daytime sleepiness (EDS) were excluded.

#### 1.2 Normal control subjects

Total 31 age-matched control subjects were consecutively recruited based on the following criteria: no neurological symptoms and signs; no systemic diseases affecting patients’ sleep; no sleep walking history; no epilepsy history; no hallucination and other psychiatric symptoms; no alcohol and drugs abuse; no essential tremor, PD, secondary parkinsonism and Parkinson plus syndrome; no obvious cognitive impairment; no systemic infectious diseases and autoimmune diseases; no intracranial diseases; no dysarthria and mental illness which affect expression, and head MR is normal.

Control subjects with blood donation histories and female controls who had not been through menopause were not included in this study. Control subjects with history of RLS, PLMD and EDS were excluded.

### 2. Clinical assessments for PD patients

#### 2.1 RBD

RBDSQ is a self-rating questionnaire with 13 items of clinical characteristics of RBD based on the International Classification of Sleep Disorder-II (ICSD-II) [18]. RBDSQ covers the symptoms of RBD and one item clarifies the presence of any neurological disease [18]. Total 6 points on RBDSQ is the best cut-off value for identifying RBD in PD patients with sensitivity of 0.842 and specificity of 0.962 [19]. PD patients with RBDSQ ≥ 6 points and < 6 points were classified into PRBD group and no PRBD (NPRBD) group, respectively. RBD assessment was performed by an interviewer blinded to the patients.

#### 2.2 Demographics information

In PD patients, gender, age, age of onset, disease duration, disease severity and LED were recorded. The severity of PD was assessed by Hoehn-Yahr (H-Y) stage. According to the method for clinical phenotypes classification by Schrag [20], participants were divided into tremor type, bradykinesia-rigidity type and mixed type of PD. Non-motor symptoms questionnaire (NMS-Quest) is a widely used [16] and self-administered 30-item instrument for screening the presence of an array of non-motor symptoms and calculating the incidence of each non-motor symptom. NMS-Quest is well accepted by PD patients [16], and detects non-motor symptoms at a higher frequency in PD patients than control subjects.

### 3. Collections of CSF and serum

Anti-parkinsonian drugs were withheld for 12–14 hours if patients’ condition allowed. Total 3 ml CSF was taken in a polypropylene tube between 7 a.m. and 10 a.m. under fasting condition through lumbar puncture. Total 2 ml venous whole blood was collected. Approximately 0.5 ml volume of CSF and serum were aliquotted into separate Nunc cryotubes and kept frozen at -80°C until ready for assay. Each aliquot dedicated for each measure to avoid freeze-thawing and protein degradation.

### 4. Detections of the levels of iron and related proteins in CSF and serum

The levels of iron and related proteins, including transferrin, ferritin and lactoferrin in CSF and serum from PD patients were detected by Enzyme Linked Immunosorbent Assay (ELISA). Ab83366 kit for iron, Ab108911 kit for transferrin, and Ab108837 kit for ferritin were from Abcam Company (Cambridge, United Kindom). E01L0224 kit for lactoferrin was from Shanghai Lanji Biological Limited Company (Shanghai, China).

### 5. Detections of the levels of inflammatory factors in CSF and serum

The levels of inflammatory factors, including NO, H_2_O_2_, L–1β, TNF-α and PGE_2_ in CSF and serum were detected.

The levels of NO and H_2_O_2_ were measured by chemical colorimetric method. A012 kit for NO and A064 kit for H_2_O_2_ were from Nanjing Jiancheng Biological Engineering Research Institute (Nanjing, China).

The levels of IL–1β, TNF-α and PGE_2_ in CSF and serum were measured by ELISA.1R040 kit for IL–1β and 1R350 kit for TNF-α were from RB Company (USA). CSB-E07965h kit for PGE_2_ was from CUSABIO Company (Wuhan, China).

### 6. Data analyses

Statistical analyses were performed with SPSS Statistics 20.0 (IBM Corporation, New York, USA). P value was statistically significant when it was less than 0.05.

Demographics information was compared between PRBD and NPRBD groups. The levels of iron and related proteins and inflammatory factors in CSF and serum were compared among control, NPRBD and PRBD groups.

Continuous variables, if they were normally distributed, were presented as means ± standard deviations and compared by ANOVA test. Bonferroni correction was performed in further comparisons between two groups. P value was significant when it was < 0.05. Continuous variables, if they were not normally distributed, were presented as median (quartile) and compared by nonparametric test. P value was significant when it was < 0.017(0.05/3) in further comparisons between two groups. Discrete variables were compared by Chi square test.

Pearson correlation analyses were made between RBDSQ score and the levels of iron and related proteins in CSF and serum, between RBDSQ score and the levels of inflammatory factors in CSF and serum, among the levels of iron, tranferrin, NO and IL–1β in CSF and age, age of disease onset, disease duration, score of UPDRS III and total number of NMS, and between the levels of iron and related proteins and inflammatory factors in CSF in PD group.

Multiple linear regression models were established, in which levels of iron and tranferrin in CSF in PD group were set as dependent variables, whereas RBDSQ score, disease duration, H-Y staging, UPDRS III score and total number of NMS were set as independent variables.

P value was significant when it was < 0.05.

## Results

### 1. Incidence of PRBD in PD patients

Sixty-seven of 210 PD patients (31.90%) have PRBD (RBDSQ score≥6 points). The average RBDSQ score of PRBD and NPRBD groups is 7.99 ± 1.64 and 2.17 ± 1.26 points, respectively. In 67 PD patients with PRBD, 25 patients (37.31%) manifest with PRBD before the onset of motor symptoms.

### 2. Demographics information and numbers of nonmotor symptoms between NPRBD and PRBD groups

Demographics information and numbers of non-motor symptoms are compared between NPRBD and PRBD groups (Tables 1, 2 and 3). The results show that PRBD group have longer disease duration, more advanced disease stage, higher score of UPDRS III, higher incidence of rigidity-bradykinesia type of PD and more nonmotor symptoms than NPRBD group.

**Table 1:** Demographics information and numbers of non-motor symptoms in NPRBD and PRBD groups.

	**NPRBD group**	**PRBD group**	**P**
Number (cases)	143	67	
Male/Total [cases/total (%)]	77/143 (53.85%)	38/67 (56.72%)	0.697
Age [years, median (quartile)]	59.59 ± 10.63	60.91 ± 11.69	0.464
Age of disease onset (years, mean ± SD)	56.60 ± 11.36	55.93 ± 11.72	0.702
Disease duration [years, median (quartile)]	2.00 (1.00~4.00)	3.00 (1.35~6.00)	**0.004** **
Hoehn-Yahr stage [stage, median (quartile)]	1.50 (1.50~2.50)	2.00 (1.50~2.50)	**0.040** *
UPDRS III [points, mean ± SD]	22.18 ± 9.271	28.59 ± 15.218	**0.005** **
Clinical phenotype [cases/total (%)]			
Tremor type	44/143 (30.77%)	14/67 (20.90%)	0.136
Rigidity-bradykinesia type	17/143 (11.89%)	16/67 (23.88%)	**0.026** *
Mixed type	82/143 (57.34%)	37/67 (55.22%)	0.773
Total numbers of NMS (cases, mean ± SD)	8.41 ± 5.27	11.17 ± 5.96	**0.005** **
Numbers of pre-MS NMS [cases, median (quartile)]	1.00 (0~3.00)	2.00 (0.00~4.50)	0.083
Numbers of post-MS NMS [cases, median (quartile)]	5.00 (2.00~10.00)	7.00 (3.25~12.00)	0.067
Levodopa equivalent dose(mg, mean ±SD)	312.87 ± 101.67	331.12 ± 87.76	0.601

*: P<0.05,

**: P<0.01.

**Table 2:** Demographics information in NPRBD and PRBD groups with CSF collected.

	**NPRBD group**	**PRBD group**	**P**
Number (cases)	51	16	
Male/Total [cases/total (%)]	23/51 (45.10%)	9/16 (56.25%)	0.112
Age [years, median (quartile)]	61.23 ± 13.18	58.87 ± 10.54	0.561
Age of disease onset (years, mean ± SD)	58.67 ± 13.31	60.14 ± 11.61	0.697
Disease duration [years, median (quartile)]	2.00 (1.00~3.00)	3.00 (1.40~6.00)	**0.013** *
Hoehn-Yahr staging [stage, median (quartile)]	1.50 (1.50~2.50)	2.00 (1.50~2.60)	**0.038** *
UPDRS III [points, mean ± SD]	24.22 ± 7.13	29.76 ± 11.35	**0.022** *
Clinical phenotype [cases/total (%)]			
Tremor type	17/51 (33.33%)	5/16 (31.25%)	0.136
Rigidity-bradykinesia type	9/51 (17.65%)	3/16 (18.75%)	0.889
Mixed type	25/51 (49.01%)	8/16 (50.00%)	0.773
Levodopa equivalent dose(mg, mean ±SD)	321.19 ± 98.61	313.32 ± 101.76	0.477

*: P<0.05.

**Table 3:** Demographics information in NPRBD and PRBD groups with serum collected.

	**NPRBD group**	**PRBD group**	**P**
Number (cases)	102	40	
Male/Total [cases/total (%)]	47/102 (46.09%)	23/40 (57.50%)	0.213
Age [years, median (quartile)]	56.31 ± 13.38	62.76 ± 9.71	0.645
Age of disease onset (years, mean ± SD)	53.86 ± 7.31	57.18 ± 13.22	0.513
Disease duration [years, median (quartile)]	2.00 (2.00~4.00)	3.00 (2.45~6.25)	**0.031** *
Hoehn-Yahr staging [stage, median (quartile)]	1.50 (1.50~2.50)	2.25 (1.89~3.00)	**0.022** *
UPDRS III [points, mean ± SD]	21.64 ± 10.31	29.76 ± 14.55	**0.017** *
Clinical phenotype [cases/total (%)]			
Tremor type	33/102 (32.35%)	11/40 (27.50%)	0.136
Rigidity-bradykinesia type	13/102 (12.75%)	12/40 (30.00%)	**0.026** *
Mixed type	56/102 (54.90%)	17/40 (42.50%)	0.089
Levodopa equivalent dose(mg, mean ±SD)	321.31 ± 115.61	325.87 ± 121.56	0.771

*: P<0.05.

### 3. Relationship among PRBD, abnormal iron metabolism and inflammation in brain

#### 3.1 Relationship between PRBD and the levels of iron and related proteins in CSF

The levels of iron, transferrin, ferritin and lactoferrin in CSF are compared among control, NPRBD and PRBD groups (Table 4). The levels of iron and transferrin in CSF in PRBD group are prominently higher than that in control and NPRBD groups. Further analyses demonstrate RBDSQ score increases with the elevated levels of iron (r = 0.343 P = 0.008) and transferrin (r = 0.329, P = 0.009) in CSF in PD group. Ferritin level in CSF in PD group is significantly decreased comparing with control group, however, is not different between PRBD group and NPRBD groups.

**Table 4:** The levels of iron and related proteins in CSF among control, NPRBD and PRBD groups.

	**Control group**	**NPRBD group**	**PRBD group**	**P** ^**1**^	**P** ^**2**^	**P** ^**3**^
Number (cases)	31	51	16			
Iron [mean ± SD] (nmol/ml)	0.495 ± 0.173	0.458 ± 0.197	0.699 ± 0.369	0.853	**0.000** **	**0.002** **
Transferrin [median (quartile)] (ug/ml)	0.153 (0.117~0.172)	0.145 (0.081~0.191)	0.262 (0.155~0.357)	0.796	**0.001** ^**#**^	**0.003** ^**#**^
Lactoferrin [median (quartile)] (ug/ml)	126.078 (64.874~207.359)	158.745 (98.655~183.699)	139.060 (106.670~207.308)	0.553	0.486	0.769
Ferritin[median (quartile)] (ng/ml)	14.583 (9.393~18.903)	6.094 (2.581~14.435)	10.900 (3.054~15.464)	0.003^**#**^	0.113	0.386

P^1^: NPRBD group vs. Control group; P^2^: PRBD group vs. Control group, P^3^: PRBD group vs. NPRBD group.

**: P<0.01.

^**#**^: P<0.017.

#### 3.2 Relationship between PRBD and the levels of inflammatory factors in CSF

The levels of inflammatory factors, including NO, H_2_O_2_, IL–1β, PGE_2_ and TNF-α in CSF are compared among control, NPRBD and PRBD groups (Table 5). The elevated levels of NO and IL–1β are found in PRBD group comparing with that in control group. The level of NO in PRBD group is strikingly enhanced comparing with that in NPRBD group. The level of IL–1β in PRBD group is elevated comparing with NPRBD group. Further analysis indicates RBDSQ score increases with the enhanced levels of NO (r = 0.442, P = 0.000) and IL–1β (r = 0.230, P = 0.035) in CSF.

**Table 5:** The levels of inflammatory factors in CSF among control, NPRBD and PRBD groups.

	**Control group**	**NPRBD Group**	**PRBD Group**	**P** ^**1**^	**P** ^**2**^	**P** ^**3**^
Number (cases)	31	51	16			
NO[mean ± SD](mmol/L)	52.496 ± 28.073	58.887 ± 31.214	103.994 ± 39.265	0.875	**0.000** **	**0.000** **
H_2_O_2_ [median(quartile)](mmol/L)	1.973 (1.184~4.004)	2.341 (1.571~3.168)	2.177 (1.837~3.659)	0.685	0.390	0.622
IL–1β [median (quartile)] (pg/ml)	9.409 (8.535~36.169)	11.122 (8.862~69.511)	67.032 (10.438~146.820)	0.125	**0.001** ^**#**^	0.028
PGE_2_ [median (quartile)] (pg/ml)	5.955 (5.205~31.045)	10.192 (5.017~16.016)	12.753 (4.876~19.634)	0.708	0.971	0.381
TNF-α [median (quartile)] (pg/ml)	48.282 (26.689~92.395)	33.185 (20.025~51.618)	33.117 (23.981~73.758)	0.075	0.237	0.541

P^1^: NPRBD group vs. Control group; P^2^: PRBD group vs. Control group, P^3^: PRBD group vs. NPRBD group.

**: P<0.01.

^**#**^: P<0.017.

#### 3.3 Relationship between the levels of iron and related proteins and inflammatory factors in CSF

Further analyses indicate that iron level increase with the levels of NO (r = 0.427, P = 0.002) and IL–1β (r = 0.560, P = 0.000) in CSF in PD group.

#### 3.4 Relationship among levels of iron and tranferrin and inflammatory factors in CSF and age, age of disease onset, disease duration, score of UPDRS III and total number of NMS in PD group

Correlation analyses among iron level in CSF and age, age of disease onset, disease duration, score of UPDRS III and total number of NMS show that iron level in CSF is positively correlated with UPDRS III score (r = 0.306, P = 0.044) and total number of NMS (r = 0.280, P = 0.038).

Correlation analyses among transferring level in CSF and age, age of disease onset, disease duration, score of UPDRS III and total number of NMS show that transferrin level in CSF is positively correlated with total number of NMS (r = 0.310, P = 0.018).

Correlation analyses among the levels of inflammatory factors in CSF and age, age of disease onset, disease duration, score of UPDRS III and total number of NMS show no significant correlations.

#### 3.5 Influencing factors of abnormal iron metabolism in PD group

Multiple linear regression model is established, in which iron level in CSF in PD group is set as dependent variable, whereas RBDSQ score, disease duration, H-Y staging, UPDRS III score and total number of NMS were set as independent variables. The results show that RBDSQ score is the only influencing factors for iron level in CSF in PD group (regression coefficient = 0.045, P = 0.017), whereas H-Y staging, UPDRS III score and total number of NMS do not enter the regression equation (S1 Table).

Another multiple linear regression model is established, in which transferrin level in CSF in PD group is set as dependent variable, whereas RBDSQ score, disease duration, H-Y staging, UPDRS III score and total number of NMS are set as independent variables. RBDSQ score is the only influencing factors for iron level in CSF in PD group (regression coefficient = 0.016, P = 0.038). Although H-Y staging, UPDRS III score and total number of NMS enter the regression equation, they fail to influence transferrin level in CSF in PD group (S2 Table).

### 4. Relationship among PRBD, the levels of iron and related proteins and inflammatory factors in peripheral system

#### 4.1 Relationship between PRBD and the levels of iron and related proteins in serum

The levels of iron, transferrin, ferritin and lactoferrin in serum are compared among control, NPRBD and PRBD groups (Table 6). The data show that transferrin level in serum in NPRBD and PRBD groups is prominently decreased comparing with control group. Moreover, transferrin level in serum in PRBD group is reduced comparing with NPRBD group. Further analyses show no relationship between RBDSQ score and the levels of iron and related proteins (P>0.05).

**Table 6:** The levels of iron and related proteins in serum among control, NPRBD and PRBD groups.

	**Control group**	**NPRBD Group**	**PRBD Group**	**P** ^**1**^	**P** ^**2**^	**P** ^**3**^
Number (cases)	31	102	40			
Iron [mean ±SD](nmol/ml)	4.192 ± 1.054	4.124 ± 1.064	3.994 ± 0.757	0.713	NS	0.224
Transferrin [median (quartile)] (ug/ml)	0.143 (0.117~0.564)	0.080 (0.069~0.286)	0.074 (0.056~0.084)	**0.000** ^**#**^	**0.000** ^**#**^	0.026
Lactoferrin [mean ± SD] (ug/ml)	67.152 ± 36.805	61.586 ± 40.068	77.467 ± 36.372	0.514	0.450	NS
Ferrtin [mean ± SD] (ng/ml)	9.402 ± 5.139	9.930 ± 4.712	10.195 ± 2.712	0.467	0.672	NS

P^1^: NPRBD group vs. Control group;

P^2^: PRBD vs. Control group,

P^3^: PRBD group vs. NPRBD group.

^**#**^: P<0.017.

#### 4.2 Relationship between PRBD and the levels of inflammatory factors in serum

The levels of inflammatory factors, including NO, H_2_O_2_, IL–1β, PGE_2_ and TNF-α in serum are compared among control, NPRBD and PRBD groups (Table 7). The elevated levels of IL–1β and PGE_2_ in serum are observed in NPRBD and PRBD groups comparing with control group. PGE_2_ level in serum in PRBD group is elevated comparing with that in NPRBD group. Further analysis shows RBDSQ score increases with enhanced PGE_2_ level (r = 0.306, P = 0.000) in serum in PD group.

**Table 7:** The levels of inflammatory factors in serum among control, NPRBD and PRBD groups.

	**Control group**	**NPRBD Group**	**PRBD group**	**P** ^**1**^	**P** ^**2**^	**P** ^**3**^
Number (cases)	31	102	40			
NO [median(quartile)](mmol/L)	44.52 6 (26.278~74.039)	60.993 (31.138~98.204)	43.750 (33.577~73.653)	0.157	0.986	0.090
H_2_O_2_ [median(quartile)](mmol/L)	27.167 (23.306~37.033)	27.453 (22.877~33.341)	28.578 (24.593~35.460)	0.878	0.576	0.464
IL–1β [median(quartile)] (pg/ml)	8.215 (7.265~9.432)	34.262 (9.527~53.584)	41.578 (9.528~128.980)	**0.000** ^**#**^	**0.000** ^**#**^	0.331
PGE_2_ [median(quartile)](pg/ml)	6.147 (3.494~9.268)	6.665 (3.697~14.574)	12.575 (5.328~16.829)	**0.006** ^**#**^	**0.000** ^**#**^	0.023
TNF-α [mean ± SD] (pg/ml)	68.918 ±14.863	62.893 ± 21.200	65.119 ± 20.071	0.922	0.499	0.815

P^1^: NPRBD group vs. Control group;

P^2^: PRBD group vs. Control group,

P^3^: PRBD group vs. NPRBD group.

^**#**^: P<0.017.

#### 4.3 Relationship between the levels of iron and related proteins and inflammatory factors in serum

Correlation analyses are made between the levels of iron and related proteins and inflammatory factors in serum. The data do not indicate any correlation between them (P>0.05).

## Discussion

In the current investigation, 31.90% of total PD patients have PRBD, supporting that RBD is a very common non-motor symptom in PD patients. Twenty-five out of 67 (37.31%) PD patients with PRBD manifest with this special type of sleep disorder prior to the appearance of motor symptoms, illustrating that RBD is one of prodromal symptoms of PD. The diagnosis of RBD requires video-polysomnographic (v-PSG) evidence of the absence of atonia during REM sleep and motor activities according to ICSD-II [18]. In this study, patients were only evaluated by RBDSQ. Although previous studies have proved that RBDSQ has high sensitivity and specificity in identifying RBD in PD patients [19], the diagnosis in this study is probable RBD, which is not containing v-PSG analysis and results. This limitation may more or less influence the accuracy of the results and decrease the power of the conclusions and speculations.

In this study, PD patients with PRBD have more advanced disease stage, severer motor symptoms and more non-motor symptoms, illustrating that these patients exhibit a mode of more serious and widespread neurodegenerative progression. Comparing with patients with tremor type of PD, those who are with rigidity type of PD exhibit severer neuronal loss in the regions that are highly relevant to RBD [2], such as locus coeruleus [21], which may partially support our finding indicating that patients with PRBD have higher frequency of rigidity-bradykinesia type of PD.

Abundant evidences indicate that excessive iron accumulation in particular brain regions is one of the underlying mechanisms of PD [21]. In the population recruited in this study, iron levels in CSF in PRBD and NPRBD group are elevated comparing with control group. Moreover, comparing with NPRBD group, iron level in CSF in PRBD group is evidently increased (Table 4), implying a potential relationship between iron and PRBD in PD patients. In addition, RBDSQ score increases with the elevated iron level in CSF in PD patients, and RBDSQ score is the only factor influencing the change of iron level in CSF in PD patients, suggesting that the appearance of RBD may impact iron accumulation in brain **(Result 3.4 and 3.5)**.

We then investigated the role of the proteins involving iron metabolism on PRBD. The result indicates that transferrin level in CSF in PRBD group is obviously enhanced comparing with NPRBD and control groups (Table 4). Moreover, RBDSQ score increases with the enhanced transferrin level in CSF in PD patients (**Result 3.1**), and RBDSQ score is the only factor influencing the change of iron level in CSF in PD patients (**Result 3.4 and 3.5**). Transferrin is the primary receptor-mediated transporter of iron from peripheral system to CNS across the blood-brain barrier [22]. In brain, iron may be transported by brain-derived transferrin, which delivers iron to neuron and glia via transferrin receptor [23]. We speculated that in the diseased brain, the balance between production of brain-derived transferrin and its clearance may be disturbed. In this study, blood-brain barrier of PRBD group may be more seriously damaged than that of NPRBD group, allowing the entry of transferrin from peripheral system to brain enormously. These results imply that dysregulation of major proteins related to iron metabolism may contribute to the development of PRBD in PD patients.

Ferritin is also a protein relating iron metabolism, and particularly important in iron-storage in brain [24]. In this study, ferritin level in CSF in NPRBD group is significantly lower than that in control group (Table 4), implying that insufficient ferritin is unable to handle the overloaded iron, and thus is associated with PD. However, there is no significant difference about ferritin level in CSF between PRBD and NPRBD groups, and between PRBD and control groups, demonstrating that ferritin may not be a major player for PRBD of PD.

Currently, neuroinflammation featured by microglial activation and related production of inflammatory factors is thought to be the engine driving dopaminergic neuron death [25]. Particularly, neuroinflammatory factors are implicated to interact with the mechanisms of sleep [26]. In a previous animal experiment, IL–1β level was increased in hypothalamus of mice with REM sleep deprivation [27], implying a potential role of neuroinflammation on sleep disorders. By far, no study focuses on the relationship between neuroinflammation and RBD in PD patients. In this study, the levels of NO and IL–1β in CSF in PRBD group are increased comparing with NPRBD and control groups (Table 5), and RBDSQ score is increased with the elevated levels of NO and IL–1β in CSF in PD group (**Result 3.2**). These results indicate a potential role of neuroinflammation on PRBD in PD patients, and NO and IL–1β may be the potential neuroinflammatory indicators for RBD in PD patients.

Peripheral iron is transferred into brain through blood-brain barrier, thus it is possible that peripheral iron may participate in the pathogenesis of PD [28]. Several studies have identified the alterations of iron metabolism-related proteins in peripheral system of PD patients [29]. In this study, the level of transferrin, a main vehicle for iron transport to control the level of free iron [30], in serum from NPRBD group is significantly decreased comparing with control group (Table 6). More importantly, transferrin level in serum in PRBD group is reduced comparing with that in NPRBD and control groups (Table 6). Hence, the elevated transferrin level in CSF in PRBD group may be partly due to its entry from peripheral to center systems, a process that highly contributes to iron accumulation in brain.

Peripheral inflammation may be related to neuroinflammation in SN [15] and neuroinflammation in SN may trigger inflammatory responses outside brain [31]. In this study, the levels of IL–1β and PGE_2_ in serum in NPRBD and PRBD groups are significantly increased comparing with control group (Table 7). Based on the fact that IL–1β level in CSF is evidently elevated in PRBD group comparing with control group (Table 5), we deduce that excessive peripheral IL–1β may enter brain and exacerbate neuroinflammation in PD brains.

Sleep has been found to be correlated with certain inflammatory factors in serum. IL–6 plays a regulatory role on normal sleep [32]. Inflammatory factors, such as PGE_2_ and IL–6 in plasma are elevated in healthy volunteers with sleep deprived [33]. In this study, PGE_2_ level in serum in PRBD group is enhanced comparing with that in NPRBD and control groups (Table 7), and RBDSQ score displays a remarkably positive correlation with PGE_2_ level in serum in PD group (**Result 4.2**). These results suggest a potential role of peripheral inflammation on RBD, and PGE_2_ may be a potential peripheral indicator for RBD in PD patients.

Previous studies have identified a tight association between inflammation and PD [34]. In an animal study, investigators have observed that microglial activation is associated with increased iron level in SN [35]. Meanwhile, dysregulation of iron may be attributable to the remarkably increased inflammatory factors generated by microglia [36]. In addition, excessive iron accumulation has been shown to lead to dopaminergic neuron death [37]. However, the relationship between inflammation and iron in PD patients with RBD is rarely investigated. In the current study, iron level exhibits positive correlation with the levels of NO and IL–1β in CSF in PD group (**Result 4.3**), indicating that more microglia may be activated by extensive iron accumulation in brain. As there is a close relationship between PRBD and iron in CSF in PD patients, we hypothesize that the enhanced iron in brain may cause continuous microglial activation and generate overwhelming inflammatory factors, leading to sustained neuronal death in RBD-related brain areas and symptoms of RBD in PD patients.

Moreover, the results show that iron level in CSF is correlated with the UPDRS III score and total number of NMS, and tranferrin level in CSF is correlated with total number of NMS. Thus, the levels of iron and tranferrin in CSF are elevated with the deterioration of motor and nonmotor symptoms in PD patients with PRBD. In PD patients with PRBD, more severe motor symptoms and more NMS are related to PRBD because more iron may accumulate in the brain with the worsening of motor symptoms and occurrence of more NMS. The elevated iron in the brain regions relevant to RBD may activate more microglia and induce severer inflammation, contributing to the degeneration of RBD-related neurons and propagation of RBD. Thus, PD patients with RBD may exhibit a mode of more extensive and severer neurodegenerative process due to the excessive iron accumulation in brain.

The frequency of PRBD in PD patients is 31.90%. PRBD group has longer disease duration, more advanced disease stage, severer motor symptoms, higher incidence of rigidity-bradykinesia type of PD and more non-motor symptoms. Excessively deposited iron is closely associated with PRBD in PD patients. The elevated iron level in the brains of PD patients with PRBD may be due to the translocation of transferrin from peripheral system to brain, causing abnormal iron metabolism. Overloaded iron-mediated neuroinflammation may serve as an underlying mechanism of PRBD in PD patients, and additionally, peripheral inflammation may contribute to the development of PRBD in PD. More severe motor and nonmotor symptoms may contribute to the propagation of RBD in PD patients because of the more extensive iron accumulation. Thus, inhibition of excessive iron-induced inflammation may be a novel target of drug development for PD patients with RBD.

## Supporting Information

S1 Table Influencing factors for iron level in CSF in PD group. (DOC)

S2 Table Influencing factors for transferrin level in CSF in PD group. (DOC)

## References

[1] Arnulf I. REM sleep behavior disorder: motor manifestations and pathophysiology. Mov disord. 2012;27:677–689. 10.1002/mds.24957 22447623

[2] Postuma RB, Gagnon JF, Vendette M, Charland K, Montplaisir J. REM sleep behaviour disorder in Parkinson's disease is associated with specific motor features. J Neurol Neurosurg Psychiatry. 2008;79:1117–1121. 10.1136/jnnp.2008.149195 18682443

[3] Romenets SR, Gagnon JF, Latreille V, Panniset M, Chouinard S, Montplaisir J, et al Rapid eye movement sleep behavior disorder and subtypes of Parkinson's disease. Mov disord. 2012;27:996–1003. 10.1002/mds.25086 22733427

[4] Gjerstad MD, Boeve B, Wentzel-Larsen T, Aarsland D, Larsen JP. Occurrence and clinical correlates of REM sleep behaviour disorder in patients with Parkinson's disease over time. J Neurol Neurosurg Psychiatry. 2008;79:387–391. 17557796 10.1136/jnnp.2007.116830

[5] Wallis LI, Paley MN, Graham JM, Grünewald RA, Wignall EL, Joy HM, et al MRI assessment of basal ganglia iron deposition in Parkinson's disease. J Magn Reson Imaging. 2008;28:1061–1067. 10.1002/jmri.21563 18972346

[6] Sofic E, Riederer P, Heinsen H, Beckmann H, Reynolds GP, Hebenstreit G, et al Increased iron (III) and total iron content in post mortem substantia nigra of parkinsonian brain. J Neural Transm. 1988;74:199–205. 3210014 10.1007/BF01244786

[7] Mariani S, Ventriglia M, Simonelli I, Donno S, Bucossi S, Vernieri F, et al Fe and Cu do not differ in Parkinson's disease: a replication study plus meta-analysis. Neurobiol aging. 2013;34:632–633. 10.1016/j.neurobiolaging.2012.05.015 22738721

[8] Hozumi I, Hasegawa T, Honda A, Ozawa K, Hayashi Y, Hashimoto K, et al Patterns of levels of biological metals in CSF differ among neurodegenerative diseases. J Neurol Sci. 2011;303:95–99 21292280 10.1016/j.jns.2011.01.003

[9] Farhoudi M, Taheraghdam A, Farid GA, Talebi M, Pashapou A, Majidi J, et al Serum iron and ferritin level in idiopathic Parkinson. Pak J Biol Sci. 2012;15:1094–1097. 24261127 10.3923/pjbs.2012.1094.1097

[10] Fellner L, Jellinger KA, Wenning GK, Stefanova N. Glial dysfunction in the pathogenesis of alpha-synucleinopathies: emerging concepts. Acta Neuropathol. 2011;121:675–693. 10.1007/s00401-011-0833-z 21562886 PMC4730553

[11] Block ML, Zecca L, Hong JS. Microglia-mediated neurotoxicity: uncovering the molecular mechanisms. Nature reviews. Neuroscience. 2007;8:57–69. 10.1038/nrn2038 17180163

[12] Zhang W, Yan ZF, Gao JH, Sun L, Huang XY, Liu Z, et al Role and mechanism of microglial activation in iron-induced selective and progressive dopaminergic neurodegeneration. Mol Neurobiol. 2014;49:1153–1165. 10.1007/s12035-013-8586-4 24277523 PMC4878835

[13] Zhang W, Phillips K, Wielgus AR, Liu J, Albertini A, Zucca FA, et al Neuromelanin activates microglia and induces degeneration of dopaminergic neurons: implications for progression of Parkinson's disease. Neurotox Res. 2011;19:63–72. 10.1007/s12640-009-9140-z 19957214 PMC3603276

[14] Lindqvist D, Hall S, Surova Y, Nielsen HM, Janelidze S, Brundin L, et al Cerebrospinal fluid inflammatory markers in Parkinson's disease–-associations with depression, fatigue, and cognitive impairment. Brain Behav Immun. 2013;33:183–189. 23911592 10.1016/j.bbi.2013.07.007

[15] Villaran RF, Espinosa-Oliva AM, Sarmiento M, De Pablos RM, Argüelles S, Delgado-Cortés MJ, et al Ulcerative colitis exacerbates lipopolysaccharide-induced damage to the nigral dopaminergic system: potential risk factor in Parkinson`s disease. J Neurochem. 2010;114:1687–1700. 10.1111/j.1471-4159.2010.06879.x 20584104

[16] Lindqvist D, Kaufman E, Brundin L, Hall S, Surova Y, Hansson O. Non-motor symptoms in patients with Parkinson's disease—correlations with inflammatory cytokines in serum. PloS one. 2012;7:e47387 10.1371/journal.pone.0047387 23082161 PMC3474801

[17] Hughes AJ, Daniel SE, Kilford L, Lees AJ. Accuracy of clinical diagnosis of idiopathic Parkinson's disease: a clinico-pathological study of 100 cases. J Neurol Neurosurg Psychiatry. 1992;55:181–184. 1564476 10.1136/jnnp.55.3.181 PMC1014720

[18] Stiasny-Kolster K, Mayer G, Schafer S, Moller JC, Heinzel-Gutenbrunner M, Oertel WH. The REM sleep behavior disorder screening questionnaire–-a new diagnostic instrument. Mov disord. 2007;22:2386–2393. 17894337 10.1002/mds.21740

[19] Nomura T, Inoue Y, Kagimura T, Uemura Y, Nakashima K. Utility of the REM sleep behavior disorder screening questionnaire (RBDSQ) in Parkinson's disease patients. Sleep Med. 2011;12:711–713. 10.1016/j.sleep.2011.01.015 21700495

[20] Schrag A, Jahanshahi M, Quinn N. What contributes to quality of life in patients with Parkinson's disease? J Neurol Neurosurg Psychiatry. 2000;69:308–312. 10945804 10.1136/jnnp.69.3.308 PMC1737100

[21] Andersen HH, Johnsen KB, Moos T. Iron deposits in the chronically inflamed central nervous system and contributes to neurodegeneration. Cell Mol Life Sci. 2014;71:1607–1622. 10.1007/s00018-013-1509-8 24218010 PMC3983878

[22] Rhodes SL, Buchanan DD, Ahmed I, Taylor KD, Loriot MA, Sinsheimer JS, et al Pooled analysis of iron-related genes in Parkinson's disease: association with transferrin. Neurobiol Dis. 2014;62:172–178. 10.1016/j.nbd.2013.09.019 24121126 PMC3968945

[23] Zheng W, Monnot AD. Regulation of brain iron and copper homeostasis by brain barrier systems: implication in neurodegenerative diseases. Pharmacol Ther. 2012;133:177–188. 10.1016/j.pharmthera.2011.10.006 22115751 PMC3268876

[24] Connor JR, Boeshore KL, Benkovic SA, Menzies SL. Isoforms of ferritin have a specific cellular distribution in the brain. J Neurosci Res. 1994;37:461–465. 8021970 10.1002/jnr.490370405

[25] Whitton PS. Inflammation as a causative factor in the aetiology of Parkinson's disease. Br J Pharmacol. 2007;150:963–976. 17339843 10.1038/sj.bjp.0707167 PMC2013918

[26] Wilson CJ, Finch CE, Cohen HJ. Cytokines and cognition–-the case for a head-to-toe inflammatory paradigm. J Am Geriatr Soc. 2002;50:2041–2056. 12473019 10.1046/j.1532-5415.2002.50619.x

[27] Kang WS, Park HJ, Chung JH, Kim JW. REM sleep deprivation increases the expression of interleukin genes in mice hypothalamus. Neurosci Lett. 2013;556:73–78. 10.1016/j.neulet.2013.09.050 24080377

[28] Logroscino G, Marder K, Graziano J, Freyer G, Slavkovich V, LoIacono N, et al Altered systemic iron metabolism in Parkinson's disease. Neurology. 1997;49:714–717. 9305329 10.1212/wnl.49.3.714

[29] Madenci G, Bilen S, Arli B, Saka M, Ak F. Serum iron, vitamin B12 and folic acid levels in Parkinson's disease. Neurochem Res. 2012;37:1436–1441. 10.1007/s11064-012-0729-x 22367474

[30] Moos T, Rosengren Nielsen T, Skjorringe T, Morgan EH. Iron trafficking inside the brain. J Neurochem. 2007;103:1730–1740. 17953660 10.1111/j.1471-4159.2007.04976.x

[31] De Pablos V, Barcia C, Martinez S, Gomez A, Ros-Bernal F, Zamarro-Parra J, et al MPTP administration increases plasma levels of acute phase proteins in non-human primates (Macaca fascicularis). Neurosci Lett. 2009;463:37–39. 10.1016/j.neulet.2009.07.069 19638294

[32] Vgontzas AN, Bixler EO, Lin HM, Prolo P, Trakada G, Chrousos GP. IL–6 and its circadian secretion in humans. Neuroimmunomodulation. 2005;12:131–140. 15905620 10.1159/000084844

[33] Haack M, Lee E, Cohen DA, Mullington JM. Activation of the prostaglandin system in response to sleep loss in healthy humans: potential mediator of increased spontaneous pain. Pain. 2009;145:136–141. 19560866 10.1016/j.pain.2009.05.029 PMC2737342

[34] Beal MF. Mitochondria, oxidative damage, and inflammation in Parkinson's disease. Ann N Y Acad Sci. 2003;991:120–131. 12846981 10.1111/j.1749-6632.2003.tb07470.x

[35] Hunter RL, Liu M, Choi DY, Cass WA, Bing G. Inflammation and age-related iron accumulation in F344 rats. Curr Aging Sci. 2008;1:112–121. 20021380 10.2174/1874609810801020112

[36] Wang J, Song N, Jiang H, Wang J, Xie J. Pro-inflammatory cytokines modulate iron regulatory protein 1 expression and iron transportation through reactive oxygen/nitrogen species production in ventral mesencephalic neurons. Biochim Biophys Acta. 2013;1832:618–625. 10.1016/j.bbadis.2013.01.021 23376588

[37] Qian ZM, Shen X. Brain iron transport and neurodegeneration. Trends Mol Med. 2001;7:103–108. 11286780 10.1016/s1471-4914(00)01910-9

