# Supplementary material for: Investigation on Abnormal Iron Metabolism and Related Inflammation in Parkinson Disease Patients with Probable RBD
Source: PLoS One. 2015 Oct 2;10(10):e0138997. doi: 10.1371/journal.pone.0138997 (PMC4592206; doi:10.1371/journal.pone.0138997)
Supplement: S1 Table — (DOC) [file pone.0138997.s001.doc]

**S1 Table** Influencing factors for iron level in CSF in PD group

|  | **B** | **Std. Error** | **P** |
| --- | --- | --- | --- |
| **Constant** | 0.376 | 0.087 | 0.000** |
| **RBDSQ score** | 0.045 | 0.018 | **0.017*** |

*: P＜0.05, **: P＜0.01
