# Supplementary material for: Investigation on Abnormal Iron Metabolism and Related Inflammation in Parkinson Disease Patients with Probable RBD
Source: PLoS One. 2015 Oct 2;10(10):e0138997. doi: 10.1371/journal.pone.0138997 (PMC4592206; doi:10.1371/journal.pone.0138997)
Supplement: S2 Table — (DOC) [file pone.0138997.s002.doc]

**S2 Table Influencing factors for transferrin level in CSF in PD group**

|  | **B** | **Std. Error** | **P** |
| --- | --- | --- | --- |
| **Constant** | 0.107 | 0.060 | 0.084 |
| **RBDSQ score** | 0.016 | 0.007 | **0.038*** |
| **Disease duration** | 0.003 | 0.008 | 0.708 |
| **H-Y staging** | 0.005 | 0.033 | 0.450 |
| **UPDRS III score** | -0.004 | 0.002 | 0.063 |
| **Number of NMS** | 0.008 | 0.004 | 0.057 |

*: P＜0.05
